# Obligations of the Public to the Medical Profession

**Published:** 1867-06

**Authors:** R. C. Word

**Affiliations:** Atlanta, Ga.


ATLANTA

Medical and Surgical Journal.

NEW SERIES.

Vol. VIII. JUNE, 1867. No. 4.

### ORIGINAL COMMUNICATIONS.

### ARTICLE I.

*Obligations of the public to the Medical Profession. Read before the Medical Association of the State of Georgia, at their last annual meeting*, by R. C. Word, M. D., of Atlanta, Ga. (Published by order of the Association.)

It is proposed in this paper to submit a few thoughts in relation to the want of appreciation, by the people, of the peculiar difficulties which, to the medical profession, more than any other, have resulted from the late unfortunate war. The cause of medical science, not less than the interests of the practitioner, is jeopardized; and it would seem, therefore, a duty, both to the public and the profession, to speak out upon this subject.

In former times, the principles of medical ethics, by which we were governed, were, in some degree, understood and acquiesed in by the more intelligent in the community. These principles recognized certain reciprocal obligations between the members of the medical profession and the public, and both were expected to perform their part; now, however, the rule appears to be changed—the physician is expected to perform his obligations, while the people seem to regard themselves released from their's.

We think it may be said, without boasting, that the *true men* of the medical profession in Georgia, and indeed throughout the whole South, are endeavoring to discharge their duties to science, and to the public, and are moving onward, despite the adverse circumstances of the times, in the same noble and benevolent spirit which has ever characterized medical men as a class.

The existence of the *Medical Association of Georgia* is, of itself, evidence of a spirit of investigation and progress amongst the members of the profession in the State. Its labors are devoted to the cause of science and to the public good.

There are two medical journals in successful operation in Georgia, and several others in the South; and the work of collecting statistics and useful facts, from the records and experience of the war, is being diligently prosecuted.

On the other hand, what can we say of the conduct of the public towards the profession? Amid the turmoil and confusion of business pursuits, and the anxieties incident to the present disturbed state of the country, men seem to have lost sight of those great principles and rules of action which look to the general welfare. One of these rules, which should govern the conduct of every good citizen, is the furtherance of science, and the advocacy of truth.

The great progress of medical science in the last half century, and the discoveries resulting therefrom, have proven of incalculable benefit to mankind. Take the single article of chloroform, and reflect upon the relief it afforded to thousands of our suffering soldiers during the late horrible war, not to mention the suffering it has prevented in private practice, and who can place an estimate on its value? In the treatment of disease, in surgery, in chemistry, in public Hygiene, and, in fact, in every department of medical science, the public is reaping the benefits of a rapid and unprecedented advancement.

Is it the desire of the people that this work of progress and improvement shall continue? If so, they should not neglect the *true men* of the profession. The sober, the educated, and the conscientious members of the profession should be sustained; for to them the country looks for the promotion of medical science, and they are the true conservators of the public health. Upon them alone can the people safely depend in cases of emergency; and their presence furnishes a safeguard and protection to life and to health which should not be lightly esteemed.

How often does it happen, especially in this day of accidents by steam and machinery, that cases occur in which the highest skill and science alone can avail to save life,—cases wherein impudence, and humbugger ycan not cloak the ignorance and inefficiency of the imposter,—and wherein the life of the sufferer absolutley depends upon the prompt attendance of the skillful and educated physician!

The presence of the intelligent medical man in a community is then a matter of serious importance; for critical cases, of the kind alluded to, may happen at any moment, and, indeed, are far more frequent than is supposed; for they are not always palpable to the non-professional observer, even in surgical cases, much less in ordinary diseases. It is in the latter class of cases that the quack so well succeeds in gulling the people who, while acknowledging the necessity of calling upon the men of science in these rare and critical cases, nevertheless too often bestow upon the quack the more lucrative, because the more frequent, emoluments of the daily practice. Such a course on the part of the people is both wrong in principle and suicidal in policy; and it is well that the public be advised of the fact, that it is driving from the profession those who are morally and intellectually best qualified to practice its humane and responsible duties.

But patronage to quackery, even in legitimate medicine, is not the only evil of which we now complain: there is yet another which is forcing many worthy men from the practice of medicine into other channels;—it is that *medical bills are not paid*; and that medical men are not allowed, by public sentiment, to present their bills for collection, and require prompt payment thereof, as is done in other departments. Custom has invested the medical profession with a dignity which places it in a different position from other avocations in the matter of collecting debts. If the physician duns his patrons he lowers himself in their estimation, and not unfrequently gives offense. Why is this, and why is it considered a lowering of professional dignity for the physician to demand his pay? The cash system now holds in every other departmert. How is the physician to meet the cash demands that are daily made upon him, when he is required to credit indefinitely any and every-body?

When the medical man applies for credit at the store, or the provision market, he finds that deference to professional dignity does not avail to relieve him from the demand of the cash system; and the merchant plainly tells him,—"Sir, my rule is cash: we let no goods go without the money." This, it will be said, is right. Grant it. But reverse the case, and let this identical merchant send for the physician, who replies to him,—"Sir, my rule is cash: I visit no case without the money." Would not the merchant be highly offended—and would he not ever afterwards regard this physician as an exacting and unfeeling man, and unworthy of patronage?

It is a fact well known, and one to which the public mind should be directed, that hundreds of the best men in the profession are being literally starved out by this unjust and ungrateful discrimination. We say ungrateful, because the conscientious medical man has claims upon the community far beyond the amount due by the few who are willing or able to compensate him for his services. He is a public benefactor in the highest sense of the word. The people are strangely insensible of the fact, that the physician does an amount of gratuitous labor far surpassing that of all other callings combined; and as the burden of careing for the poor should rest equally upon all classes, the undue proportion which the physician sustains should be placed to his credit as against the community. The argument that these charities are incident to the profession he has chosen, and must therefore be borne, is too illiberal and unjust to merit a reply. Yet it is evident, that such is the light in which their services are viewed, and that little or no merit is attached to their performance. To such an extent, indeed, has this feeling grown upon the people's mind, that the physician seems to be regarded as a mere philanthropist, whose duty and pleasure it is to act for the public, and who requires, and is entitled to no compensation.

In Germany, and in other European Countries, the Government provides for the medical treatment of the poor; but here, the Legislature not only refuses to compensate the practitioner, but heightens the infliction by imposing upon him a heavy specific tax.

In 1860, the Medical Association of Georgia, in a memorial to the Legislature, in reference to the injustice of the specific professional tax, thus alludes to the gratuitous services of the physician:

"At all seasons, and in all kinds of weather, in the dark hours of night, when others are asleep, the medical man passes from one scene of distress to another, bestowing his labor, impairing his health, and dispensing drugs to the indigent sick."

To this course he is impelled by two powerful forces; the first and greatest, is the demand of humanity, which, to a concientious man, leaves often no alternative by which to escape the call. The second, is the force of public sentiment, which will not tolerate in the physician that freedom of action, which it allows to others. The merchant may refuse credit to whom he chooses; the druggist may decline to sell to an insolvent customer, and it is well; but the physician who exercises this liberty, brings upon himself the severest censure, and consequent injury to his character and business.

To the many cases of casuality and death which occur in this fast age, a large proportion of which is amongst the poorer classes, the physician stands a ready servant, subject to every beck and call, and is expected and required to have in readiness all the appliances and material, at whatever cost, adapted to every emergency. By his promptness, skill, and benevolent agency, he relieves large numbers, and oft'-times rescues them from impending death. When under analogous circumstances, a party is snatched from a burning dwelling, or a watery grave, the individual who performs the deed, is esteemed a hero. When a mariner rushes to the rescue of a distressed crew, he gains for himself laurels of praise, and medals of honor. Not so, the physician. He is regarded as having performed a mere commonplace duty, and scarcely meets with a passing commendation; and such is the tyranny of custom and law, that if he refuses to respond to every call, he encounters the indignant frown of the community, and failing, from want of facilities or other cause, to adopt the most scientific treatment, he becomes liable to prosecution and heavy damages.

When the Cholera, or other destructive malady rages as an epidemic in a community, the physician remains at his post, facing danger and death for the public. In medico-legal investigations, at coroners' inquests, and in post mortem inspections, the services of the physician are required, and yet the State has made no adequate provision for his compensation.

If in the days of peace and prosperity, these burdens bore unequally and hard upon the medical man, what are they now, when the proportion of poor to be treated has increased ten fold, when the proceeds of the negro practice have been cut off, and when specific taxes by the State, the county, and the Federal authorities, are extorted from the practitioner?

Medical men, as a class, are proverbially benevolent and kind, and have ever borne with patience the heavy responsibilities of the practice and the exactions of the public; but the time has arrived, when, in consequence of their own desdestitution, and the impoverished condition of the masses, they feel constrained to protest against the vast inequality of the burdens they are called upon to bear.

Other facts could be adduced in proof of the positions assumed, and to show that the physician is a public benefactor, and is entitled to the gratitude and support, instead of the censure and neglect of the public. But we conclude by recapitulating the points we have endeavored to establish.

1st—There are reciprocal obligations between the public and the members of the medical profession.

2d—Medical men are nobly discharging their duties; but the people are unmindful of their obligations which require them, both as a matter of principle and of policy, to support the true men of the profession.

3d—Medical men are not paid: they are forced to adopt the old credit system, which is but little better than starvation, as the result of which, many good men are leaving the profession.

4th—Medical men are benefactors to the public, for which neither the Legislature nor the masses of the people have any just appreciation.

